# Exploring Oxylipins in Host–Microbe Interactions and Their Impact on Infection and Immunity

**DOI:** 10.3390/cimb47030190

**Published:** 2025-03-14

**Authors:** Robert J. Neff, Christopher D. Radka

**Affiliations:** Department of Microbiology, Immunology, and Molecular Genetics, University of Kentucky, Lexington, KY 40536, USA; robert.neff@uky.edu

**Keywords:** lipids, immune modulation, eicosanoids, prostaglandins, virulence, signaling

## Abstract

Plasma lipids are essential components of biological systems, transported through interactions with proteins to maintain cellular functions. These lipids exist in various forms, such as fatty acids, glycerolipids, glycerophospholipids, sphingolipids, sterols, and prenol lipids, derived from dietary intake, adipose tissue, and biosynthesis. While the association between certain fatty acids and cardiovascular diseases has been widely recognized, polyunsaturated fatty acids (PUFAs) exhibit cardioprotective effects, reducing risks of arrhythmias and heart-related mortality. This is due to their role in the production of eicosanoids, which modulate inflammation. Chronic inflammation, particularly in obesity, is significantly influenced by fatty acids, with saturated fatty acids promoting inflammation and PUFAs mitigating it. Oxylipins, bioactive molecules derived from the oxidation of PUFAs, play crucial roles in immune regulation across various organisms, including plants, fungi, and bacteria. These molecules, such as prostaglandins, leukotrienes, and resolvins, regulate immune responses during infection and inflammation. The production of oxylipins extends beyond mammals, with fungi and bacteria synthesizing these molecules to modulate immune responses, promoting both defense and pathogenesis. This review delves into the multifaceted effects of oxylipins, exploring their impact on host and microbial interactions, with a focus on their potential for therapeutic applications in modulating infection and immune response.

## 1. Introduction

Plasma lipids play a critical role in the body, primarily relying on their interactions with proteins to maintain their dispersion and solubility within the bloodstream. These lipids are classified into distinct groups based on their chemical properties, with simple fatty acids and similar compounds typically binding to albumin [1]. In contrast, more complex lipids are transported by specialized plasma lipoproteins, ensuring their effective distribution and function [2]. Mammals contain nearly 600 different lipid molecules, distributed across six major lipid categories: fatty acids, glycerolipids, glycerophospholipids, sphingolipids, sterols, and prenol lipids [3,4]. The fatty acids that serve as building blocks for these complex lipids originate from a combination of dietary intake, the breakdown of adipose tissue, and endogenous biosynthesis [5].

Notably, while saturated and monounsaturated fatty acids have been linked to increased risks of arrhythmias and sudden cardiac death, polyunsaturated fatty acids (PUFAs) seem to exert a protective effect against cardiovascular disease, reducing the risk of cardiac-related mortality [6,7,8]. This protective role is largely attributed to the eicosanoids produced from PUFAs, such as prostaglandins and leukotrienes, which actively participate in mitigating inflammatory responses [9]. Chronic inflammation, a common hallmark of conditions like obesity, is significantly influenced by fatty acids, with growing evidence suggesting that free fatty acids play a central role in regulating these inflammatory processes. While saturated fatty acids exacerbate the inflammatory effects of lipopolysaccharides, certain PUFAs seem to have the opposite effect, potentially dampening inflammation through their oxidized metabolites [5].

**Figure 1:**
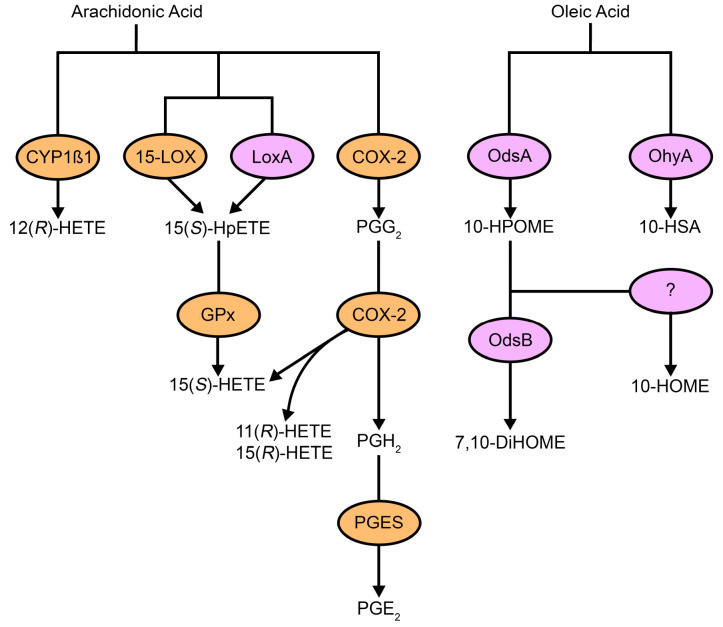
Host and microbial pathways for oxylipin production. Host cells and pathogens use unsaturated fatty acid substrates, such as arachidonic acid and oleic acid, to produce oxylipins. Some oxylipins are made by both prokaryotic (*purple*) and eukaryotic (*orange*) enzymes, while others are specific to one or the other. Host-derived fatty acids also contribute to microbial pathways. Abbreviations: CYP1β1, cytochrome P450 family 1 subfamily B member 1; 15-LOX, arachidonate 15-lipoxygenase; LoxA, secretable arachidonate 15-lipoxygenase; COX-2, cyclooxygenase 2; Gpx, glutathione peroxidase; PGES, prostaglandin E synthase; OdsA, oxylipin-dependent quorum-sensing system A; OdsB, oxylipin-dependent quorum-sensing system B; OhyA, oleate hydratase; HETE, hydroxyeicosatetraenoic acid; HpETE, hydroperoxyeicosatetraenoic acid; PG, prostaglandin; HPOME, hydroperoxyoctadecenoic acid; HSA, hydroxystearic acid; DiHOME, dihydroxyoctadecenoic acid; HOME, hydroxyoctadecenoic acid.

Oxylipins—bioactive molecules derived from the oxidation of PUFAs—serve as essential signaling compounds across a wide range of organisms, from prokaryotes to eukaryotes [10,11]. These molecules include a variety of bioactive compounds, such as eicosanoids (prostaglandins, leukotrienes, and thromboxanes) and resolvins. In plants, for example, oxylipins like jasmonic acid are central to regulating growth and defense mechanisms [12], much as eicosanoids regulate immune responses in mammals. Among mammals, the oxidation of arachidonic acid (AA) is one of the most extensively studied pathways, leading to the synthesis of a wide array of eicosanoids that regulate immune function and inflammation. AA is stored in membrane phospholipids and is released at elevated levels upon cell activation, where it is metabolized into bioactive mediators like prostaglandins and leukotrienes through a series of specific enzymatic pathways [13,14]. These enzymes, known as oxygenases, are divided into three primary classes: cyclooxygenases (COXs), lipoxygenases (LOXs), and monooxygenases [15]. Together, they facilitate the conversion of PUFAs into various oxidized products (Figure 1), although alternative radical-driven pathways also contribute to oxylipin production [16]. Both the COX and LOX pathways are well-established routes in the metabolism of AA, and research has shown that the production of oxylipins is not exclusive to mammals; plants, bacteria, and fungi also produce these molecules, suggesting that they play a widespread and evolutionarily conserved role in communication and regulation, from immunity to developmental processes.

Interestingly, the production of oxylipins is not limited to mammals. Pathogenic microbes, including bacteria and fungi, also synthesize oxylipins, either from exogenous fatty acids or through de novo pathways, to manipulate host immune responses and promote infection. This review aims to address several key gaps in the current understanding of oxylipins in microbial pathogenesis. Specifically, we will provide evidence that both pathogenic fungi and bacteria produce oxylipins and demonstrate the critical role of these oxylipins in pathogenesis. Microbial oxylipins closely resemble host-derived oxylipins, suggesting that pathogen-derived oxylipins may mimic host molecules to help pathogens regulate immune responses. By exploring these points, we aim to highlight the potential of oxylipins as novel therapeutic targets in the treatment of infectious diseases.

## 2. Oxylipin Immunomodulation

Oxylipins are integral mediators in the regulation of inflammatory responses during both injury and infection [17]. One of the most studied groups of oxylipins, prostaglandins, are locally produced lipids that can either promote or resolve inflammation, depending on the specific context, the type of cell involved, and the anatomical site of action. Inflammatory responses often lead to hyperalgesia, an enhanced sensitivity to pain, which serves as a hallmark symptom of inflammation [18]. Prostaglandins E_2_ (PGE_2_) [19] and I_2_ [20] have been particularly implicated in the potentiation of thermal hyperalgesia in experimental mouse models. These prostaglandins enhance the activation of the capsaicin receptor, a key player in the sensation of pain [21]. Macrophages, a central component of the immune system, produce these prostaglandins in response to pro-inflammatory signals, such as bacterial lipopolysaccharides. Interestingly, reverse-transcription polymerase chain reaction experiments have shown that PGE_2_ not only induces the production of pro-inflammatory cytokines like tumor necrosis factor alpha (TNF-α), interleukin 1 alpha (IL-1α), and IL-6 but also exerts a regulatory effect by suppressing the production of other cytokines, such as IL-1β and IL-8 [22,23]. This dual functionality underscores the complex nature of prostaglandins in mediating inflammation.

PGE_2_ plays complex and often contradictory roles in immune responses, influencing various conditions such as cancer, autoimmune diseases, and infections. In cancer, PGE_2_ suppresses antitumor immunity by inhibiting the expansion and differentiation of CD8+ T cells within the tumor microenvironment through EP2/EP4 signaling [24,25]. This inhibition promotes immune evasion and tumor progression. However, blocking PGE_2_ signaling can restore T-cell function and enhance anticancer immunity [25], suggesting that targeting PGE_2_ may offer therapeutic benefits. In autoimmune diseases, PGE_2_ contributes to the expansion and activation of T_h_17 cells, which play a role in conditions such as multiple sclerosis and rheumatoid arthritis [26,27]. Despite this, PGE_2_ also has a regulatory role, suppressing T_h_1 responses and promoting the resolution of inflammation, which may help restore tissue homeostasis and prevent further damage in autoimmune disorders [26,28]. During infections, PGE_2_ can dampen the host’s immune response, as seen in diseases like tuberculosis, by suppressing innate immunity and facilitating pathogen survival [29,30]. On the other hand, PGE_2_ can also play a protective role by promoting anti-inflammatory macrophage phenotypes, helping to limit excessive inflammation and preventing tissue damage during infection [30,31].

The mechanistic basis for how PGE_2_ exhibits both pro-inflammatory and anti-inflammatory effects depends on its interaction with four receptor subtypes (EP1-EP4). These receptors can mediate opposite effects, with the concentration of PGE_2_ and the type of cell involved determining the outcome. For instance, PGE_2_ promotes inflammation through several mechanisms. It enhances vascular permeability and promotes leukocyte infiltration, both of which contribute to inflammation [32,33]. It also activates mast cells through the EP3 receptor [33], worsening the inflammatory response. Additionally, PGE_2_ can inhibit regulatory T cells (Tregs) through EP4, activating the immune system to respond to the microbiota and fostering intestinal inflammation [34]. At low concentrations, PGE_2_ stimulates pro-inflammatory cytokine production by activation of nuclear factor-kappa B (NF-κB) [31].

However, PGE_2_ also has anti-inflammatory effects. It can suppress Type 2 inflammation by inhibiting eosinophil chemotaxis and limiting T_h_2 cell activation [35,36]. At higher concentrations, PGE_2_ inhibits NF-κB activity, reducing pro-inflammatory cytokine production and promoting the resolution of inflammation [31]. Furthermore, PGE_2_ induces the development of FOXP3+ regulatory T cells through the EP4 receptor, contributing to immune suppression and tissue homeostasis.

The impact of PGE_2_ extends beyond pain modulation; it also significantly influences immune responses. For instance, exposure to PGE_2_ has been shown to promote the migration of Langerhans cells—specialized dendritic cells found in the skin—to regional lymph nodes. Although this migration enhances the T-cell stimulatory capacity of Langerhans cells, it also simultaneously suppresses CD4+ T-cell proliferation [37], highlighting the nuanced effects of PGE_2_ on the immune system. Building on this reasoning, parasite-derived prostaglandin D_2_ has been observed to activate epidermal Langerhans cells in the context of skin infections [38]. This activation causes their retention within the epidermis, impairing their ability to migrate to other sites. As a result, the skin’s contact hypersensitivity response is diminished, which helps limit excessive inflammation.

Interestingly, in a guinea pig model of contact dermatitis, PGE_2_ is produced on the skin upon exposure to allergens [39], further linking prostaglandins to skin immune responses. Additionally, a human keratinocyte skin cell line produces leukotriene B_4_, as well as 12- and 15-HETE, when exposed to bacterial *Actinobacilli* species in vitro [40]. Supernatant from this skin cell line after bacterial exposure significantly enhances the production of PGE_2_ and other oxylipins by neutrophils compared to neutrophils stimulated with supernatants from unexposed skin cells [40]. These findings underscore the role of oxylipins in modulating immune responses during bacterial skin infections and inflammation.

Beyond prostaglandins, other oxylipins such as hydroxy fatty acids, including 9- and 13-HODE (hydroxyoctadecadienoic acid), are byproducts of linoleic acid peroxidation and are found to accumulate in inflammatory disease states. In patients with progressive rheumatoid arthritis, for instance, 9-HODE levels in low-density lipoprotein particles increase significantly [41]. Northern blotting assays have revealed that 9-HODE induces IL-1β mRNA expression in human macrophages [42]. Additionally, enzyme-linked immunosorbent assays confirm that both 9-HODE and 13-HODE can stimulate the release of IL-1β cytokines from these cells, further emphasizing the impact of these bioactive lipids on the inflammatory cascade.

This section underscores the complexity and versatility of oxylipins, particularly prostaglandins and hydroxylated fatty acids, in modulating immune responses. Their ability to both promote and resolve inflammation, depending on the context, highlights their importance as central players in the regulation of immune function. The ongoing research into these molecules continues to reveal their diverse functions in various pathological conditions, offering potential therapeutic avenues for managing chronic inflammatory diseases.

## 3. Fungal Oxylipins

**Figure 2:**
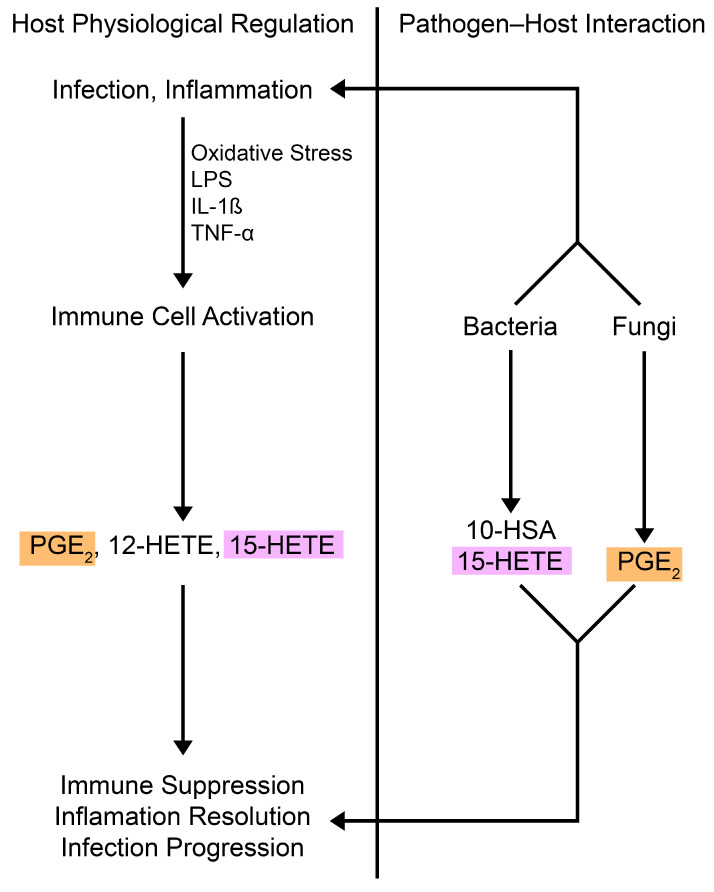
Oxylipins in microbial pathogenesis and host signaling. Host immune cells produce oxylipins like PGE_2_, which promote immunosuppressive effects, and 15-HETE, which regulates immune responses. The lipid mediators are actively secreted during infection or inflammation, balancing pro- and anti-inflammatory factors [28,29,51,52,53]. Pathogens (e.g., *Pseudomonas aeruginosa*, *Staphylococcus aureus*, and *Candida albicans*) produce and use oxylipins to manipulate host immune responses, mimicking host regulation. PGE_2_ (*orange*) is produced by fungi and host cells, while 15-HETE (*purple*) is made by bacteria and host cells. Abbreviations: LPS, liposaccharide; IL-1β, interleukin 1 beta; TNF-α, tumor necrosis factor alpha; PG, prostaglandin; HETE, hydroxyeicosatetraenoic acid; HSA, hydroxystearic acid.

Most fungi do not naturally produce significant quantities of AA in the way that mammals do, so they acquire environmental AA and convert it into various oxidized products through enzymatic pathways [43,44]. Additionally, fungi synthesize oxylipins common to both plants and animals, such as 9(*S*)-HODE and 13(*S*)-HODE, which are produced by the LOX pathway [45]. This ability to synthesize oxylipins is shared between fungi and mammals, with both groups utilizing eicosanoids—such as prostaglandins and leukotrienes—as key mediators in immune modulation and other physiological processes [45,46,47,48] (Figure 1 and Figure 2).

Fungal pathogens, particularly those involved in human infections, often manipulate oxylipin production to their advantage, using these molecules to regulate host immune responses and promote infection. For example, *Candida albicans*, a fungus normally present in the human microbiota, can become pathogenic under certain conditions, leading to life-threatening infections [49]. The immune system generally restricts the overgrowth of *C. albicans* in the intestines, but the fungus can circumvent these defenses by producing eicosanoids like PGE_2_. In the presence of human keratinocytes, *C. albicans* and *C. tropicalis* produce significantly higher levels of PGE_2_, which suppresses the expression of interferon γ-inducible protein 10 (IP-10), a molecule involved in the inflammatory response [50]. This suppression of IP-10 may impair the body’s ability to mount an effective response to a cutaneous fungal infection.

Prostaglandins, particularly PGE_2_, play a critical role in modulating the immune response during infection. By suppressing T_h_1-type immune responses while promoting a T_h_2-type response [54,55,56,57], PGE_2_ helps establish a favorable environment for the pathogen, thereby limiting the ability of macrophages to clear *C. albicans* and other fungal species [58,59]. Interestingly, although *C. albicans* lacks a COX homolog, nonspecific synthetic oxygenase inhibitors, but not COX-specific synthetic inhibitors, can block PGE_2_ production in the fungus [60]. This suggests that *C. albicans* may utilize alternative enzymatic pathways to produce prostaglandins, possibly through enzymes like stearyl-coenzyme A desaturase (Ole2) and multicopper ferroxidase (Fet3) [60]. Ole2 does not directly affect AA or prostaglandins; however, it likely alters upstream fatty acids, supplying substrates or intermediates used for PGE_2_ synthesis [60,61]. Fet3 may regulate iron homeostasis, affecting enzymes involved in PGE_2_ synthesis [61], and mutants deficient in Fet3 have reduced PGE_2_ levels [60,61]. The deletion of these enzymes does not completely abolish PGE_2_ production [60,61], indicating additional pathways exist in fungal prostaglandin biosynthesis. Importantly, the absence of Ole2 impairs *C. albicans*’ ability to colonize the gastrointestinal tract and makes the fungus more susceptible to macrophage phagocytosis [61], underscoring the impact of oxylipins on virulence [62]. Ole2 and Fet3 are key components of a unique fungal pathway for prostaglandin synthesis, though their roles appear to be indirect or regulatory rather than catalytic. Further research is needed to clarify this pathway. Additionally, PGE_2_ signaling suppresses CD4+ T-cell activation in the lamina propria and the development of colitis in a mouse model of dextran sodium sulfate-induced colitis [63]. This anti-inflammatory mechanism may help explain, at least in part, how *Candida* species can colonize the gut, as it dampens the immune response, allowing the fungus to thrive in the gastrointestinal environment.

In addition to prostaglandins, *C. albicans* also synthesizes resolvin E_1_ (RvE_1_), an anti-inflammatory oxylipin that reduces neutrophil migration in response to IL-8 [64]. RvE1 plays a complex role in fungal infections, and its biosynthesis is blocked by LOX and monooxygenase inhibitors. In a mouse model of systemic candidiasis, RvE_1_ enhances neutrophil phagocytosis and promotes the production of reactive oxygen species [64], which are critical for fungal clearance. However, the impact of RvE_1_ on fungal virulence appears to be dose-dependent, with low concentrations of RvE_1_ conferring protection, and high concentrations potentially driving an excessive immune response.

The regulation of RvE_1_ involves selective receptor interactions, immune cell modulation, and context-dependent signaling. RvE_1_ primarily acts through two receptors: ChemR23 and BLT1. As an agonist for ChemR23 on macrophages and dendritic cells, RvE_1_ promotes anti-inflammatory and pro-resolving effects by reducing cytokine production and enhancing phagocytosis [65,66]. In contrast, RvE_1_ antagonizes BLT1, a receptor for leukotriene B_4_ (LTB_4_), to suppress neutrophil recruitment and actin polymerization, key processes in inflammation [65,66]. RvE_1_ also modulates cytokine levels, inhibiting pro-inflammatory cytokines like IL-6, IL-17, and IL-23, while boosting anti-inflammatory cytokines such as TGF-β. In dendritic cells and T_h_17 cells, RvE_1_ prevents T_h_17 differentiation and reduces their migration by downregulating CCR6 expression [65,67]. Additionally, RvE_1_ limits dendritic cell motility by inhibiting LTB_4_-BLT1 signaling [65], essential for actin polymerization and migration, thus reducing dendritic cell-driven T-cell activation in immune responses. RvE_1_’s role in inflammation resolution is evident in its ability to enhance microbial clearance and prevent excessive tissue damage. For instance, it reduces neutrophil infiltration and inflammatory mediators like IL-1β and MCP-1 in acute lung injury models [68]. Through these mechanisms, RvE_1_ balances immune responses, resolving inflammation while maintaining tissue homeostasis. Its effects are context-dependent, shaped by receptor expression, cellular targets, and inflammatory signals.

Pathogenic yeasts such as *C. neoformans* also produce oxylipins, including leukotrienes and prostaglandins, even in the absence of exogenous AA supplementation. *C. neoformans* produces a dehydrogenated form of PGE_2_, known as 15-keto-PGE_2_, which activates the host nuclear receptor peroxisome proliferator-activated receptor gamma (PPARγ), promoting intracellular survival during macrophage infection [69]. Inhibition of 15-keto-PGE_2_ production impairs fungal survival, emphasizing the importance of oxylipin-mediated immune modulation in fungal pathogenesis.

In addition to these effects, fungal prostaglandins have been implicated in regulating the yeast-to-hyphal transition in *C. albicans* and *C. neoformans* [70]. This transition is crucial for fungal virulence, as it allows the fungi to disseminate throughout the body, invade tissues, and form biofilms [71,72]. In the yeast form, these fungi can adhere to endothelial cells and spread throughout the bloodstream [73,74,75]. PGE_2_ produced during hyphal formation modulates host immunity by reducing pro-inflammatory cytokines like TNF-α and increasing anti-inflammatory cytokines like IL-10, aiding fungal evasion and persistence [70,72]. However, in the hyphal form, they become more adept at evading the host immune system and infiltrating tissues [76]. COX inhibitors like aspirin block hyphal formation and biofilm development in *C. albicans* [72,77], and mutants that are unable to undergo this transition exhibit significantly reduced infectivity [78,79,80], further supporting the critical effect of oxylipin-mediated signaling in fungal virulence. Thus, the fungus synthesizes PGE_2_ from AA, typically sourced from host lipids, to utilize host resources for regulating its morphogenesis and survival [61,70].

In *Aspergillus* species, such as *Aspergillus nidulans* and *A. fumigatus*, oxylipins also play a key role in fungal pathogenesis. These species encode three COX orthologs (*ppoABC*) that contribute to prostaglandin biosynthesis [81]. In a mouse model of invasive pulmonary aspergillosis, a triple knockout mutant lacking these COX orthologs demonstrated increased resistance to oxidative stress and hypervirulence. Interestingly, prostaglandins in these fungi have different effects, with PGE_2_ inhibiting *A. nidulans* asexual sporulation (important for fungal dissemination) and PGE_2_ also inhibiting pigment formation in *A. fumigatus* hyphae. Although spore pigmentation and gliotoxin production are known to be key virulence factors in *A. fumigatus* [82,83,84], neither of these traits is affected in the *ppo* knockout mutant. This suggests that prostaglandin biosynthesis may contribute to virulence through an additional, yet unidentified, mechanism. These observations highlight the complexity of oxylipin signaling in fungal pathogens, suggesting that prostaglandins may modulate fungal growth and virulence in species-specific ways.

## 4. Bacterial Oxylipins

Bacteria, much like plants, animals, and fungi, produce oxylipins and oxylipin-like molecules that serve as essential signaling molecules, modulating a variety of physiological processes within both the bacteria themselves and their host organisms (Figure 1 and Figure 2). These molecules are critical in regulating bacterial growth, host interactions, and immune responses, often influencing the progression of infections. One well-studied example is *Pseudomonas aeruginosa*, a Gram-negative opportunistic pathogen commonly involved in mixed infections with *C albicans* [85]. It is a major contributor to morbidity and mortality in individuals with cystic fibrosis or those who are immunocompromised [86,87,88].

*P. aeruginosa* is capable of synthesizing oxylipins through the incorporation of host-derived AA. Using its LOX ortholog, LoxA, *P. aeruginosa* produces 15(*R*)-hydroxyeicosatetraenoic acid (15(*R*)-HETE), a key oxylipin that influences immune modulation [89,90,91]. Interestingly, 15(*R*)-HETE plays an important role in inducing anti-inflammatory effects in the host by promoting the synthesis of lipoxins, which are known to facilitate the resolution of inflammation [89,92]. Neutrophils, once they internalize 15(*R*)-HETE, convert this molecule into the *R* configuration of lipoxins (e.g., 15-epi-LXA_4_), which actively resolve inflammation and aid in the restoration of tissue homeostasis [93]. In contrast, the *S* stereoisomer of 15-HETE, which is naturally found in mammals, is converted into a less potent form of lipoxin (e.g., LXA_4_) that has a shorter half-life and a diminished anti-inflammatory effect [94,95]. There is evidence that *P. aeruginosa* is capable of synthesizing prostaglandins [96], but more work is needed to determine their precise impact on pathogenesis. COX-like enzymes, identified in bacterial systems, play a role in PGE_2_ production. While specific enzymes analogous to mammalian cyclooxygenases have not been fully characterized in *P. aeruginosa*, studies suggest that bacterial cyclooxygenase-like enzymes contribute to PGE_2_ synthesis [97]. This ability to modulate inflammation through the synthesis of oxylipins demonstrates how bacteria have evolved to manipulate host immune responses in ways that facilitate their survival and pathogenesis.

The presence of lipoxygenases in bacteria was first identified with the discovery of the *loxA* gene in *P. aeruginosa*. Since then, lipoxygenases have been characterized in other bacterial species including *Nitrosomonas europaea* and *Anabaena* sp. strain PCC 7120, neither of which are known to be infectious [89]. Nonpathogenic bacteria use oxylipins as autoinducers in quorum sensing to regulate gene expression based on cell density [98]. This system, distinct from traditional quorum sensing mechanisms, involves LysR-type transcriptional regulators. Oxylipins also influence bacterial motility, biofilm formation, and multicellular behavior, enhancing survival under stress [99]. Additionally, they enable bacteria to interact with plants and fungi, impacting host defense mechanisms or symbiotic relationships. For instance, bacterial oxylipins can mimic plant signaling molecules to modulate plant immunity [100]. By regulating transitions between planktonic and biofilm states, oxylipins help bacteria adapt to harsh environments [100,101], underscoring their role in bacterial communication, even in nonpathogenic species. In mammals, lipoxins like 15-HETE have important functions as signaling molecules in immune regulation. Observations demonstrate that *P. aeruginosa* secretes these oxylipins into the periplasm and extracellular spaces; however, further research is needed to fully understand the implications of this process and its impact on bacterial pathogenesis.

*P. aeruginosa* also produces other oxylipins, such as 10(*S*)-hydroxy-(8*E*)-octadecenoic acid (10(*S*)-HOME) and 7*S*,10*S*-dihydroxy-(8*E*)-octadecenoic acid (7,10-DiHOME) [98,99]. These molecules are synthesized by 10-*S* Dioxygenase (OdsA) and 7,10-Diol Synthase (OdsB), which are encoded by the PA2077 and PA2078 genes, respectively [102]. Both genes are part of an operon that also includes PA2076, which encodes OdsR, a probable transcriptional regulator. The discovery of this oxylipin biosynthetic pathway highlights a novel quorum-sensing system, known as the ODS (oxylipin-dependent quorum sensing system), that regulates bacterial motility and biofilm formation [98]. Production of 10(*S*)-HOME and 7,10-DiHOME involves secretion of OdsAB through the Xcp Type II secretion system [103], which is a distinctive feature compared to other quorum-sensing systems. These processes are essential for *P. aeruginosa* to establish persistent infections, particularly in cystic fibrosis patients where biofilms play a significant role in chronic lung infections [99,104]. Quorum sensing is a mechanism by which bacteria communicate with each other to coordinate group behaviors, such as the formation of biofilms, which increase bacterial resistance to host immune responses and antimicrobial treatments [105]. This process is driven by oxylipins derived from host oleic acid, which are essential for effective biofilm formation and pathogenicity [103]. By producing these oxylipins, *P. aeruginosa* can regulate its own gene expression, promoting biofilm formation and improving survival in hostile environments [98,99].

In vitro studies revealed that these oxylipins promote microcolony formation both over abiotic surfaces and biotic surfaces, including monolayers of A549 human alveolar epithelial cells, which *P. aeruginosa* infects in cystic fibrosis patients [106]. Furthermore, in vivo testing in *Drosophila melanogaster* showed that a *P. aeruginosa* strain with knockout mutations of both OdsA and OdsB had half the mortality rate of the wild-type strain when inoculated using the pricking method [99]. Further investigations revealed that *P. aeruginosa* senses the presence of 10(*S*)-HOME and 7,10-DiHOME based on cell density using the ODS system. These oxylipins induce the expression of their own biosynthetic operon [98]. When extracellular levels of oxylipins reach a certain threshold, they bind to OdsR, which upregulates the production of more OdsA and OdsB enzymes. These enzymes then convert additional PUFAs into oxylipins that are subsequently detected by an unknown receptor within the cell [98]. This feedback loop ensures that the quorum-sensing system is activated in response to rising oxylipin concentrations. Notably, the system requires the presence of exogenous oleic acid to activate OdsR, ensuring that the ODS system is triggered only in environments containing this fatty acid, which is typically found in living organisms. This suggests that *P. aeruginosa* has evolved to utilize host-derived oleic acid as a strategy for establishing successful infections [107]. The discovery of oxylipin production and its significance in bacterial quorum sensing, along with the identification of homologs for OdsA and OdsB across various *Pseudomonas* species and other bacterial genera, suggests that targeting oxylipin-mediated communication could offer new therapeutic opportunities to disrupt bacterial quorum sensing and biofilm formation, potentially providing novel treatments for chronic infections.

Beyond *P. aeruginosa*, other bacterial pathogens also utilize oxylipins in their interactions with hosts. *Staphylococcus aureus*, a Gram-positive bacterium commonly associated with skin infections and a frequent co-colonizer with *C. albicans* in cystic fibrosis patients [108,109], also exploits oxylipins to modulate immune responses. *S. aureus* can metabolize host-derived oleic acid into 10(*R*)-hydroxystearic acid (10-HSA) [110], a hydroxylated fatty acid that plays a crucial role in modulating immune responses during infection. Increased levels of oleic acid are correlated with more severe cystic fibrosis disease progression [111], and *S. aureus* exploits this by producing 10-HSA. This molecule activates PPARα, which suppresses the host’s innate immune response, facilitating immune evasion and enhancing the bacterium’s survival [112].

In addition, 10-HSA is abundant in the pulmonary discharge from cystic fibrosis patients infected with *P. aeruginosa* or *S. aureus* [113], providing further evidence of its impact on infection. *P. aeruginosa* may produce 10-HSA [114] through at least two distinct pathways: the LoxA enzyme and the OdsAB system. In contrast, *S. aureus* relies solely on the enzyme oleate hydratase (OhyA) to convert oleic acid from the membrane bilayer into 10-HSA [115,116,117,118]. OhyA is involved in a larger metabolic program to metabolize environmental fatty acids [119], but genetic disruption of *ohyA* does not significantly affect *S. aureus* growth in vitro [110]. Nevertheless, *S. aureus*’s ability to produce 10-HSA remains crucial for its virulence, as evidenced by the impaired infection capability in a mouse model when *ohyA* is disrupted [120]. These findings underscore the importance of oxylipin production in bacterial pathogenesis.

Furthermore, *Streptococcus pyogenes*, a bacterium responsible for flesh-eating disease, also encodes the OhyA enzyme and produces hydroxylated fatty acids, such as 10-HSA. In *S. pyogenes*, the production of these fatty acids enhances bacterial virulence by promoting adherence to host tissues and facilitating internalization into host cells. A knockout strain of *S. pyogenes* that cannot produce these hydroxylated fatty acids exhibits reduced virulence, as it is less capable of adhering to and internalizing into host keratinocytes [121]. These findings suggest that bacterial production of oxylipins not only helps modulate the immune system but also enhances pathogen fitness by facilitating critical processes such as adherence, internalization, and evasion of immune detection. Interestingly, many bacterial taxa, including probiotic beneficial symbionts from the gut microbiome, also encode the enzyme OhyA, which plays a role in oxylipin metabolism [122,123,124,125]. This suggests that OhyA may contribute to host tolerance of the gut microbiome and support symbiosis.

Another notable bacterium, *Mycobacterium tuberculosis*, a highly pathogenic bacterium responsible for tuberculosis, also manipulates the balance of oxylipins to escape immune detection and modulate the host’s inflammatory response. One of the key oxylipins involved in this process is PGE_2_, which is induced by *M. tuberculosis* during infection. PGE_2_ plays a dual role in modulating the immune response, as it suppresses pro-inflammatory cytokine production while simultaneously promoting autophagy in infected cells [29,126]. This autophagic response helps to contain the bacteria within the host cell, thereby limiting the spread of infection. However, *M. tuberculosis* also induces the synthesis of lipoxin A_4_ (LXA_4_), which triggers necrosis of infected cells, enabling the bacteria to escape and disseminate throughout the host [127,128]. This complex interplay between PGE_2_ and LXA_4_ influences the outcome of the infection, with excessive LXA_4_ promoting bacterial survival and dissemination through necrotic cell death [129,130]. Thus, the balance of oxylipins plays a critical role in determining the success of *M. tuberculosis* infection and the progression of tuberculosis.

## 5. Targeting Oxylipins in Therapy

The role of oxylipins in microbial colonization and infection remains unclear. In particular, it is uncertain how many distinct microbes rely on oxylipins for these processes. Another unresolved question in polymicrobial settings is whether all members of the microbial community need to produce oxylipins, or if production by just a few members can protect the entire community.

Several therapeutic strategies aim to target oxylipins by modulating their production, signaling pathways, or downstream effects in order to treat infections and inflammatory diseases. These strategies range from broad-spectrum approaches to highly targeted ones. For example, oxylipin biosynthesis can be inhibited using COX and LOX inhibitors. Nonsteroidal anti-inflammatory drugs (e.g., aspirin) inhibit COX enzymes, reducing prostaglandin production in both the host and pathogens like *C. albicans* [131,132]. LOX inhibitors can block bacterial exploitation of host-derived oxylipins, as seen in *P. aeruginosa* and the plant pathogen *Xylella fastidiosa* [100,133]. Another approach involves blocking oxylipin signaling through EP receptor antagonists. For example, blocking receptors like EP4 has been shown to reduce prostaglandin-mediated immunosuppression in cancer and infections [131,132], thus enhancing immune responses. Additionally, synthetic oxylipin analogs can be used to disrupt signaling. Modified oxylipins inspired by natural products have been developed to inhibit biofilm formation in pathogens like *S. aureus* by disrupting quorum sensing and biofilm matrix production [134].

A major concern in the use of broad-spectrum antimicrobial treatments is the collateral damage to the microbiome. Recent advancements in narrow-spectrum therapeutics have shown promise in creating pathogen-specific therapies [135]. One example is inhibiting *P. aeruginosa* diol synthase enzymes to block the synthesis of virulence-enhancing oxylipins like 10-HOME and 7,10-DiHOME [99,136]. Another approach that may help preserve the microbiome involves targeting oxylipins through diet. Supplementation with omega-3 fatty acids alters the oxylipin profile, favoring anti-inflammatory mediators such as resolvins, which counteract pro-inflammatory bacterial lipids [131,132]. Modifying gut microbiota through diet or probiotics can also influence oxylipin production and reduce inflammation-related diseases [132].

However, translating oxylipin-targeting therapies into clinical practice faces several challenges. Analytical and technical difficulties related to standardization, lipid complexity, and rapid turnover pose significant obstacles. Oxylipins are metabolically unstable with short half-lives, making it challenging to accurately measure their levels and effects in vivo [137]. Furthermore, variability in sample collection, preparation, and analysis across laboratories complicates the generation of reproducible data. To overcome these challenges, harmonized protocols and reference materials are essential for establishing consistent oxylipin profiles [137,138].

The complexity of oxylipin signaling pathways, which involve overlapping and redundant functions, makes it difficult to isolate their specific roles. Biological variability also adds complexity, as oxylipins can have pro-inflammatory or anti-inflammatory effects depending on tissue type, disease state, or concentration [138,139]. This variability makes therapeutic targeting difficult. Genetic, metabolic, and environmental factors also influence oxylipin production and response [138], highlighting the need for personalized approaches to treatment. Many oxylipins interact with multiple receptors or pathways, which increases the risk of unintended side effects, such as impaired platelet function or excessive immune suppression [137,140]. Moreover, developing stable formulations that effectively deliver oxylipin-modulating agents to target tissues without causing systemic side effects remains a challenge [137].

Despite these hurdles, targeting oxylipins holds significant potential for treating inflammatory diseases, infections, and cardiovascular conditions. Although few clinical trials have tested oxylipin-targeted therapies, soluble epoxide hydrolase inhibitors for cardiovascular diseases show promise and warrant further validation in diverse populations [141]. To overcome the challenges in oxylipin-based therapies, standardized protocols for oxylipin profiling must be developed. Additionally, large-scale cohort studies are needed to validate biomarkers and therapeutic targets, and advances in drug delivery systems and bioinformatics tools are necessary to enhance the precision of oxylipin-based treatments.

## 6. Conclusions

Oxylipins, bioactive lipids derived from polyunsaturated fatty acids, play critical roles in modulating immune responses, inflammation, and microbial pathogenesis in both fungi and bacteria. These molecules are involved in host–pathogen interactions, influencing microbial virulence, biofilm formation, immune evasion, and host immune regulation. In fungi, oxylipins like prostaglandins and resolvins help pathogens such as *C. albicans* and *A. fumigatus* manipulate immune responses and promote infection persistence. Similarly, bacteria such as *P. aeruginosa* and *S. aureus* exploit oxylipins to enhance pathogenicity, while beneficial gut microbiota also contribute to the oxylipin pool.

The therapeutic potential of targeting oxylipins is significant, with strategies ranging from inhibiting biosynthesis pathways (e.g., COX and LOX inhibitors) to modulating signaling through receptor antagonists and synthetic analogs. However, challenges remain in translating these strategies into clinical practice due to issues such as metabolic instability, biological variability, and potential side effects.

Future research should focus on overcoming these challenges by developing standardized protocols for oxylipin profiling, improving drug delivery systems and creating more precise narrow-spectrum treatments to avoid disrupting the microbiome. Additionally, investigating oxylipin metabolism in the gut microbiome could offer new avenues for managing intestinal dysbiosis and enhancing microbiome health. Further studies into the concentration-dependent effects of oxylipins and their interactions with multiple microbial species will be crucial in refining therapeutic approaches and identifying reliable biomarkers for infection diagnosis and treatment outcomes.
